# Invasive Group B Streptococcal Infections in Finland: A Population-based Study

**DOI:** 10.3201/eid0904.020481

**Published:** 2003-04

**Authors:** Outi Lyytikäinen, J. Pekka Nuorti, Erja Halmesmäki, Petteri Carlson, Jukka Uotila, Risto Vuento, Tapio Ranta, Hannu Sarkkinen, Martti Ämmälä, Anja Kostiala, Anna-Liisa Järvenpää

**Affiliations:** * National Public Health Institute, Helsinki, Finland; † Helsinki University Central Hospital, Helsinki, Finland; ‡ Tampere University Hospital, Tampere, Finland; § Päijät-Häme Central Hospital, Lahti, Finland; ¶ Jorvi Hospital, Espoo, Finland

**Keywords:** Group B streptococcus, invasive bacterial infection, epidemiology, population-based study, early-onset neonatal disease, prevention, research

## Abstract

We analyzed surveillance data on group B streptococcus (GBS) infection in Finland from 1995 to 2000 and reviewed neonatal cases of early-onset GBS infection in selected hospitals in 1999 to 2000. From 1995 to 2000, 853 cases were reported (annual incidence 2.2–3.0/100,000 population). We found 32–38 neonatal cases of early-onset GBS disease per year (annual incidence 0.6–0.7/1,000 live births). In five hospitals, 35% of 26 neonatal cases of early-onset GBS infection had at least one risk factor: prolonged rupture of membranes, preterm delivery, or intrapartum fever. Five of eight mothers screened for GBS were colonized. In one case, disease developed despite intrapartum chemoprophylaxis. Although the incidence of early-onset GBS disease in Finland is relatively low, some geographic variation exists, and current prevention practices are suboptimal. Establishing national guidelines to prevent perinatal GBS is likely to reduce the incidence of the disease.

Group B streptococcus (GBS), a leading cause of invasive bacterial infections in newborns, also affects pregnant women and elderly persons (*1*–*4*). In the United States, several studies have reported the incidence of GBS infection in different demographic groups, and guidelines were developed and implemented for the prevention of neonatal infection in the 1990s (*1*–*6*). In European countries, however, few population-based data on GBS infection are available and no national guidelines have been published (*7*–*10*).

In the United States, the recommended strategies to prevent perinatal GBS disease include either a risk-based or screening-based approach (*5*). In the risk-based approach, women in labor who have risk factors for GBS transmission (e.g., fever, prolonged rupture of the membranes, or preterm delivery) are offered intrapartum chemoprophylaxis. In the screening-based approach, vaginal and rectal combined swabs are cultured from all pregnant women and tested for GBS carriage during 35 to 37 weeks’ gestation. Those identified as GBS carriers are offered intrapartum chemoprophylaxis.

In Finland, laboratory-based surveillance for invasive bacterial infections, including GBS, began in 1995. To identify opportunities for prevention, we analyzed national GBS surveillance data from 1995 to 2000. To assess the proportion of cases that might have been prevented by using the risk-based or screening approaches, we reviewed birth histories of infants with early-onset GBS disease in five hospitals participating in a nosocomial infection surveillance network from 1999 to 2000. We also conducted two national surveys: one evaluating the microbiologic methods used to screen for GBS cultures in Finnish clinical microbiology laboratories and the other on current practices related to GBS screening and antibiotic use in Finnish hospitals with obstetric services.

## Methods

### Surveillance

Finnish clinical microbiology laboratories routinely notify the National Infectious Disease Registry of bacterial isolations from blood and cerebrospinal fluid. Each report includes the following information: isolation date, birth date, sex, specimen type, and treatment location. Multiple reports of the same case are combined in the database if they are received within 3 months of first isolation. A case is defined as isolation of GBS from blood or cerebrospinal fluid; early-onset neonatal disease is defined as that occurring in infants <7 days old and late-onset disease as that occurring in infants 7–89 days old.

### Additional Data Collection and Chart Review

Neonatal GBS cases that occurred in five hospitals from 1999 to 2000 were identified through hospital wide surveillance of nosocomial bloodstream infections in connection with the Finnish Hospital Infection Program (SIRO). We obtained data on deliveries, local guidelines for perinatal GBS prevention, and microbiologic data (e.g., screening method, number of specimens examined, and number of GBS-positive specimens). In cases of early-onset disease, the following data were abstracted from the medical records: prenatal GBS screening, intrapartum fever >38°C, prolonged rupture of membranes >18 h before delivery, preterm delivery at <37 weeks of gestation, receipt of intrapartum antibiotics, and outcome of illness.

### Calculation of Incidence Rates and Statistical Analysis

Data from the National Population Registry, including live births, from 1995 to 2000 were used as denominators to calculate age- and sex-specific incidence rates and early-onset and late-onset neonatal disease rates. The average annual incidences during the surveillance period were calculated by using the total number of cases, population, and live births from 1995 to 2000. To evaluate trends, rates of GBS disease in different age and sex groups were calculated for each 6-month period from January 1995 to December 2000. Data were analyzed by using Epi Info software, version 6.04 (Centers for Disease Control and Prevention, Atlanta, GA) and SAS software, version 8.2 (SAS Institute, Inc., Cary, NC). A Poisson regression model was used to assess whether the observed changes in the rates were statistically significant.

### Surveys

In February 2002, we sent structured questionnaires by electronic mail to 20 of the 28 Finnish clinical microbiology laboratories and by regular mail to all Finnish hospitals with obstetric services (n=38). The laboratories were asked about their methods for screening cultures for GBS and the hospitals about their GBS prevention policies.

## Results

From 1995 to 2000, a total of 853 cases of invasive GBS disease were identified. Of bacterial isolates, 96% were obtained from blood and 4% from cerebrospinal fluid. The average annual incidence was 2.8 cases per 100,000 population (range by year, 2.2–3.0) and varied from 1.8 to 4.0 by health district. In women aged >65 years of age, the incidence increased significantly, from 1.1 per 100,000 in 1995 to 8.2 per 100,000 in 2000 (p<0.01 by Poisson regression). No trends were identified in other age or sex groups.

Infants of <1 year of age had the highest rate and accounted for 272 (32%) of 853 of all GBS infections (Table 1); 211 (78%) of 272 were early-onset disease and 203 (96%) of 211 were identified during the first 2 days of life. The average annual incidence of early-onset infections was 0.6 per 1,000 live births (range by year, 0.6–0.7; 32–38 cases/y; Table 2) and varied from 0.1 to 1.3 by health district. In 7 of 20 health districts in the country, the average annual incidence was >0.7 per 1,000 live births. Among 211 early-onset cases, 98% of isolates were obtained from blood and 2% from cerebrospinal fluid. The average annualized incidence of late-onset infections was 0.2 per 1,000 live births (range by year, 0.1–0.3; 6–16 cases/y; Table 2) and varied from 0.0 to 0.4 by health district. Among 56 cases of late-onset GBS disease, 59% of isolates were obtained from blood and 41% from cerebrospinal fluid.

**Table 1:** Incidence of invasive group B streptococcus infection by age and sex, Finland, 1995–2000

Age group (y)	Men		Women		Total	
	No. of cases	Rate^a^	No. of cases	Rate^a^	No. of cases	Rate^a^
<1	143	79.3	129	74.7	272	77.1
1–14	1	0.04	5	0.2	6	0.1
15–64	128	1.2	193	1.9	321	1.6
>64	103	6.0	151	5.4	254	5.6
All	375	2.5	478	3.0	853	2.8

^a^Average annual incidence (cases per 100,000 population)

**Table 2:** Annual incidence of early-onset and late-onset invasive group B streptococcus infections, Finland, 1995–2000

	1995	1996	1997	1998	1999	2000
**Early-onset disease**						
No. of cases	37	37	34	38	33	32
Incidence^a^	0.6	0.6	0.6	0.7	0.6	0.6
**Late-onset disease**						
No. of cases	8	16	6	10	9	7
Incidence^a^	0.1	0.3	0.1	0.2	0.2	0.1

^a^Cases per 1,000 live births.

From 1999 to 2000, a total of 38,687 women delivered babies in the five study hospitals, accounting for one third of all live births in Finland. Of the deliveries, 20% were cesarean sections and 7% preterm deliveries. None of the hospitals had a policy for universal maternal screening of GBS. Their protocol included screening risk groups only and prescribing intrapartum prophylaxis for GBS-positive women. Patients who had previously delivered infants with GBS disease and who had tested positive for GBS bacteriuria during pregnancy were also screened. Two hospitals prescribed ampicillin, and three hospitals prescribed penicillin. To identify GBS carriers, four hospitals cultured the samples and one used an antigen test. Only vaginal swabs were collected. A total of 9,220 screening specimens were obtained; 12% of them were positive for GBS. The proportion of positive specimens varied from 4% to 21% in the five hospitals.

In the study hospitals, 26 cases of early-onset disease (0.7/1,000 live births) and four cases of late-onset disease (0.1/1,000 live births) were identified. One premature neonate died. Delivery occurred at 25 weeks of gestation, and the screening result was negative. Of 26 women who had infants with early-onset disease, 1 received intrapartum antibiotics because of prolonged rupture of the membranes and a positive screening result. Of the 25 women who did not receive intrapartum antibiotics, 18 (72%) were not screened. Eight (32%) developed at least one risk factor (six had duration of ruptured membranes >18 hours, two had delivery at <37 weeks of gestation, and one had intrapartum fever). Of the 18 women not screened, 4 (22%) showed risk factors at the time of labor (3 had duration of ruptured membranes >18 hours, and 1 had delivery at <37 weeks of gestation).

All 26 isolates from case of early-onset infection and 4 from cases of late-onset infection were evaluated for antibiotic susceptibility. All isolates were susceptible to penicillin; two isolates (8%) were resistant/intermediate to erythromycin, and one isolate (4%) was resistant to clindamycin.

### Surveys

All 20 microbiology laboratories responded. Of the laboratories, 13 (65%) had a specific laboratory request for GBS culture; 9 laboratories also requested cultures from vagina (69%) and 8 (62%) from cervix. None of the laboratories recommended rectal cultures. One laboratory used selective broth media to culture GBS.

All directors of the 38 hospitals with obstetric services responded. Written GBS prevention protocols existed in 30 (79%) hospitals. Most used a combination of risk-based and screening-based strategies; one routinely screened all pregnant women for prenatal GBS carriage. Recommendations for obstetric risk groups include screening patients for GBS when they have one of the following: premature delivery (87%), rupture of membranes without labor (82%), previous delivery of an infant with invasive GBS disease (79%), GBS bacteriuria (66%), and maternal fever during labor (53%). GBS specimens were usually obtained from the vagina (82%) or cervix (45%). No rectal cultures were taken. Culture was used to detect GBS in 82% of laboratories and antigen test in 34%. In 61% of the hospitals, chemoprophylaxis was given to all identified GBS carriers; the remaining 39% of hospitals required the presence of at least one additional obstetric risk factor before prescribing chemoprophylaxis (Table 3). When screening cultures were not performed or the results were not available at labor, chemoprophylaxis was most often given to risk groups with the following obstetric risks: intrapartum fever, previous delivery of an infant with invasive GBS disease, or prolonged rupture of membranes. Intrapartum chemoprophylaxis was given parenterally in 87% of hospitals and orally in 11%. Penicillin was recommended in 69%, cephalosporins in 19%, and aminopenicillins in 11% of hospitals.

**Table 3:** High risk groups for whom intrapartum antibiotic prophylaxis is recommended in 38 Finnish hospitals with obstetric services, 2002^a^

Risk group	No. of hospitals (%)	
	GBS specimen taken/ result positive^b^	GBS specimen not taken/results unknown
GBS-positive mothers	23 (61)	–
GBS bacteriuria during current pregnancy	15 (39)	15 (39)
Invasive GBS disease in previously delivered child	25 (66)	25 (66)
Delivery <37 wk gestation	18 (47)	9 (24)
Rupture of membranes >18 h	26 (68)	19 (50)
Intrapartum fever >38°C	31 (82)	33 (87)

^a^GBS, group B streptococcus.

^b^Only one hospital routinely screened all pregnant women for prenatal GBS carriage.

## Discussion

Compared with rates previously reported from European countries, the incidence of early-onset GBS disease in Finland is relatively low (*7*–*14*). However, the incidence is twice as high as rates reported among white infants in the United States (*6*). Data from the study hospitals in Finland also indicate that most mothers of infants with early-onset disease did not receive intrapartum antibiotics.

In Europe, most studies documenting the occurrence of early-onset GBS disease during the past decade involved a single hospital (10,12–14). European population-based data from Norway in 2001 showed an incidence of 1 case per 1,000 live births (*8*). During the period of our surveillance, the incidence of early-onset and late-onset infection in Finland remained unchanged and comparable to rates in a previous nationwide study conducted from 1985 to 1994 (early-onset disease 0.62/1,000 live births; late-onset disease 0.13/1,000 live births) (*15*). The annual number of cases of early-onset disease appears low, but surveillance is limited to culture-confirmed cases of invasive disease. The number of newborns in whom GBS is treated empirically may therefore be larger.

We also identified considerable variation in rates of early-onset infection by health district. In Finland, the need for effective preventive measures was already emphasized during the 1980s, when GBS was identified as the most important etiologic agent of neonatal septicemia (*16*). The efficacy of intrapartum chemoprophylaxis has also been demonstrated by a Finnish study (*17*). However, the prophylaxis was only introduced to heavily colonized patients detected by the streptolatex test.

In our review of 26 cases of early-onset GBS disease, 1 case-patient received intrapartum antibiotics; 31% were screened prenatally for GBS, and 35% had a risk factor evident at the time of labor. Most case-patients were not screened and had no risk factors at the time of labor; of those not screened, four later developed a risk factor. Screening was performed for those in risk groups; some patients, such as those who had previously delivered infants with GBS disease or who tested positive for GBS bacteriuria during pregnancy, were unnecessarily screened. In addition, the site where cultures were taken and isolation method used differed from those recommended (*5*,*18*–*22*).

In the United States, the decline in the incidence of GBS disease in newborns coincided with the implementation of consensus guidelines for the prevention of perinatal GBS disease beginning in 1996 (*1*). From 1993 to1998, the incidence of GBS declined 65% from 1.7 to 0.6 per 1,000 live births. Data from 1998 to 1999 indicate a further decline in incidence in selected surveillance areas. Among certain demographic groups, such as white infants, the rate has declined to 0.3 per 1,000 live births (*6*). A recent review of >300 cases of early-onset infection from the United States also showed missed opportunities for prevention, including cases that would not have been prevented even with perfect implementation of prophylaxis strategies: 21% of cases occurred despite administration of intrapartum antibiotics, 35% of case-patients had been screened perinatally for GBS, and 44% had a risk factor evident at the time labor (*2*,*6*). Forty percent of case-patients showed none of the risk-based criteria for prophylaxis unless they were screened for GBS.

GBS infection in adults in Finland accounted for 67% of the total cases. The average annualized incidence of invasive GBS infection in adults varied from 1.6 to 5.6 per 100,000 population by age group. Although the incidence among elderly women was slightly lower than in elderly men, this number appears to be increasing. This finding is similar to previous population-based incidence data reported in 1989 to 1990 from metropolitan Atlanta, Georgia, where 48% of the total GBS cases were in adults; the annual incidence was 6.2 per 100,000 (*3*). Because the Atlanta study focused on nonpregnant adults and our study did not have information on the pregnancy status of the patients, age-specific rates cannot be compared. Recent U.S. data that included pregnant and nonpregnant adults indicate a marked increase in rates among adults, particularly in elderly persons and those with underlying illness, and vary between 2.1 to 21.9 per 100,000 by age group (*4*). The reasons for differences in rates of GBS disease between countries may include demographic differences, socioeconomic factors, and variations in clinical practices, such as the frequency of taking blood cultures in diagnostic examinations. Another suggested independent risk factor for both early-onset and late-onset GBS infection in neonates is being of black race (*3*,*4*). However, the association may also be linked to socioeconomic factors. The increasing prevalence of diabetes mellitus and other underlying conditions may contribute to the increasing rates of GBS infection in adults.

A common concern in the risk-based prevention approach is that a large number of women would receive unnecessary antibiotics. Widespread use of antibiotics can lead to an increase in allergic reactions, emergence of resistant strains, and cases of antibiotic colitis. The use of intrapartum antibiotics in the United States has doubled from 1996 to 1999, coinciding with GBS prevention implementation (*23*). We were unable to obtain information on how widely prophylaxis is currently used in Finland because data on type, dose, and time of administration of intrapartum antibiotics are not documented in hospital databases. Unnecessary antibiotic use could be reduced by not offering GBS prophylaxis to women who are not carriers. A recent study suggested that screening may be more effective in prevention than the risk-based approach (*23*). Screening reaches a broader population, and persons who are screened are more likely to receive prophylactic antibiotics. However, wide-scale screening for GBS colonization may be difficult to implement.

The results of our study should be used to develop and implement national guidelines for prevention of perinatal GBS. Such guidelines would standardize prevention practices, rationalize the use of intrapartum antibiotics, and reduce the incidence of perinatal GBS disease. Further studies should be done to investigate the reasons for incidence increase among elderly women.
